# Applying a deep convolutional neural network to monitor the lateral spread response during microvascular surgery for hemifacial spasm

**DOI:** 10.1371/journal.pone.0276378

**Published:** 2022-11-02

**Authors:** Minsoo Kim, Sang-Ku Park, Yasuhiro Kubota, Seunghoon Lee, Kwan Park, Doo-Sik Kong

**Affiliations:** 1 Department of Neurosurgery, Gangneung Asan Hospital, Gangneung, Korea; 2 Department of Neurosurgery, Samsung Medical Center, Sungkyunkwan University School of Medicine, Seoul, Korea; 3 Department of Medicine, Graduate School, Yonsei University College of Medicine, Seoul, Korea; 4 Department of Neurosurgery, Konkuk University Medical Center, Konkuk University School of Medicine, Seoul, Korea; 5 Independent Researcher, Tokyo, Japan; Universiti Malaysia Pahang, MALAYSIA

## Abstract

**Background:**

Intraoperative neurophysiological monitoring is essential in neurosurgical procedures. In this study, we built and evaluated the performance of a deep neural network in differentiating between the presence and absence of a lateral spread response, which provides critical information during microvascular decompression surgery for the treatment of hemifacial spasm using intraoperatively acquired electromyography images.

**Methods and findings:**

A total of 3,674 image screenshots of monitoring devices from 50 patients were prepared, preprocessed, and then adopted into training and validation sets. A deep neural network was constructed using current-standard, off-the-shelf tools. The neural network correctly differentiated 50 test images (accuracy, 100%; area under the curve, 0.96) collected from 25 patients whose data were never exposed to the neural network during training or validation. The accuracy of the network was equivalent to that of the neuromonitoring technologists (*p* = 0.3013) and higher than that of neurosurgeons experienced in hemifacial spasm (*p* < 0.0001). Heatmaps obtained to highlight the key region of interest achieved a level similar to that of trained human professionals. Provisional clinical application showed that the neural network was preferable as an auxiliary tool.

**Conclusions:**

A deep neural network trained on a dataset of intraoperatively collected electromyography data could classify the presence and absence of the lateral spread response with equivalent performance to human professionals. Well-designated applications based upon the neural network may provide useful auxiliary tools for surgical teams during operations.

## Introduction

Hemifacial spasm (HFS) is a neurological disorder characterized by irregular and involuntary contractions of the facial muscles innervated by the ipsilateral facial nerve. The pathogenesis of HFS may involve vascular compression of the facial nerve at its origin near the brainstem, resulting in demyelination and ephaptic transmission [1, 2]. Although underdiagnosis, misdiagnosis, and the absence of population-based data obscure the prevalence of HFS, its incidence is reportedly 9.8–11 per 100,000 individuals. Microvascular decompression that detaches the culprit vessel from the facial nerve is widely accepted as the first-line surgical treatment and provides relief from spasm in more than 90% of patients [3–7].

The lateral spread response (LSR), a pathological response observed in patients with HFS and can be detected by electromyography (EMG), is elicited by the stimulation of one branch of the facial nerves, resulting in the contraction of the facial muscles innervated by the other branch of the facial nerve. Although the exact mechanism of the LSR is not fully understood, cross-transmission of antidromic activity from the stimulated branch of the facial nerve may contribute to the phenomenon. Continuous monitoring of the LSR during surgery can identify the culprit vessels and confirm that adequate decompression has been carried out [8, 9]. Such continuous monitoring requires dedicated, experienced personnel and cannot be carried out without specialized electrophysiology knowledge.

In the current study, we constructed a dataset of intraoperative EMG with expert annotation to indicate the presence or absence of the LSR. We then developed a deep neural network to classify the LSR using current-standard, off-the-shelf tools. A computer-assisted program based on the neural network could easily be adapted into a commercially available intraoperative monitoring (IOM) system. To the best of our knowledge, no previous studies have used deep learning technology with a computer vision algorithm to automate the diagnosis of LSR. If a deep learning-based algorithm could interpret IOM findings as accurately as a human professional, concerning surgical findings could be detected earlier and improve clinical outcomes.

## Methods

### Standard protocol approval, registration, and patient consent

Data acquisition and processing in this prospective study were approved by the institutional review board of Samsung Medical Center (SMC2020-10-174). The study was conducted in accordance with the relevant guidelines and regulations. The requirement of written informed consent from the participants was waived because the study involved secondary use of data that were collected primarily for clinical reasons and were appropriately de-identified.

### Image dataset

We collected LSR data from adult participants who had undergone microvascular decompression to treat HFS at our institution between August and October 2019. All data were obtained during surgery via screenshots of the IOM suite (Xltek Protektor32 IOM; Natus Neurology, Ontario, Canada). A total of 3,674 images from 50 patients were included for model training and validation: 1,869 with LSR and 1,805 without LSR. Fifty images collected from another 25 patients were used for model testing: 25 with LSR and 25 without LSR. We obtained multiple images from all participants by taking screenshots every minute during the surgical procedures.

**Fig 1:**
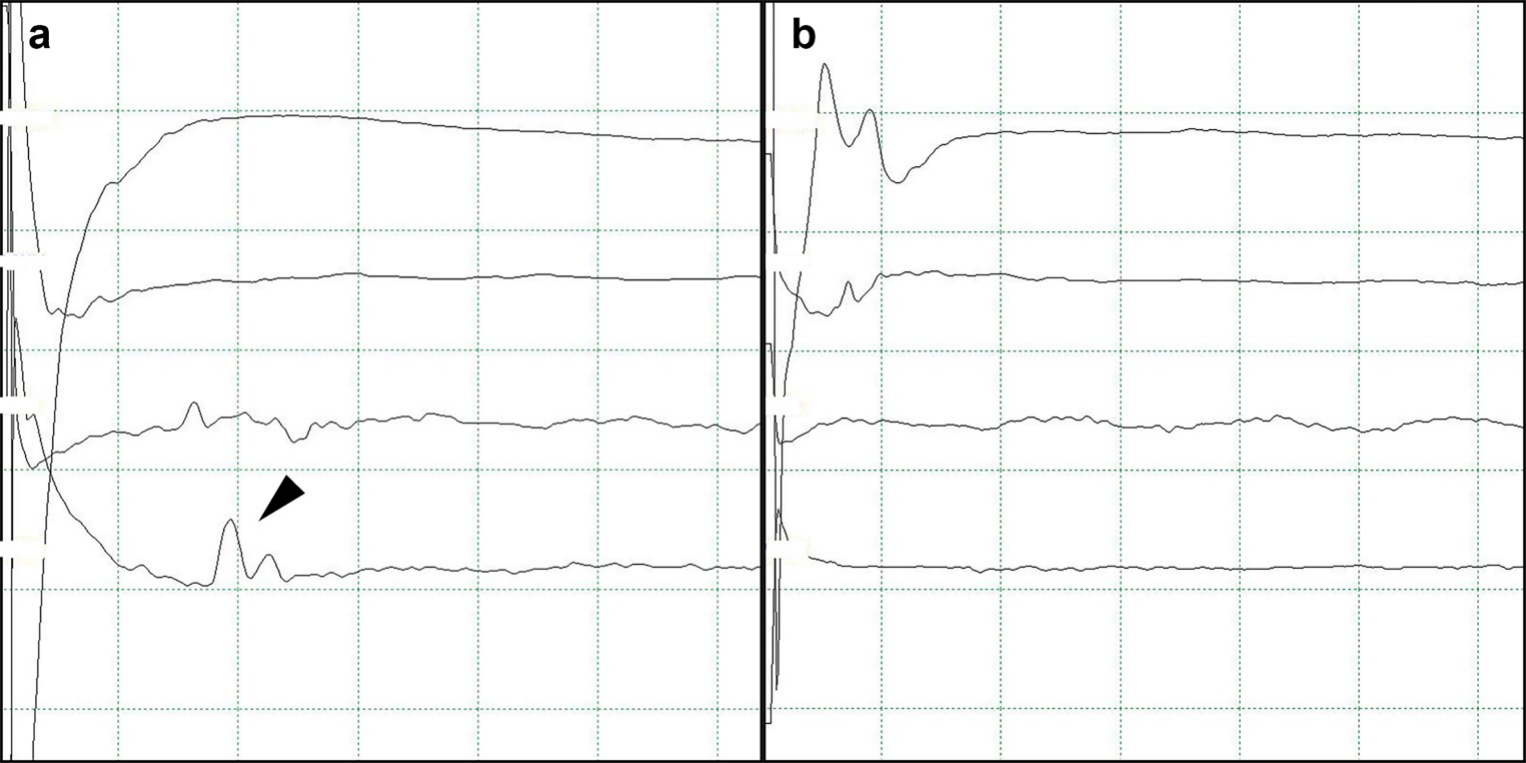
Representative images for the lateral spread response (LSR). The typical delayed electrical activity (A, LSR presence) in the mentalis muscle following stimulation of the temporal branch of the facial nerve disappeared (B, LSR absence) when the culprit vessel was detached from the facial nerve during the surgical procedure.

To maintain data consistency, anesthetic techniques, surgical procedures, perioperative patient care, and IOM were performed according to the institution’s standard protocol, which has been described elsewhere [10]. All images were annotated as either “LSR presence” or “LSR absence” by an IOM technologist (S.K.P.), confirmed by a neurosurgeon (M.K.), and then anonymized. Representative images of each LSR class are presented in Fig 1.

### Image preparation

**Fig 2:**
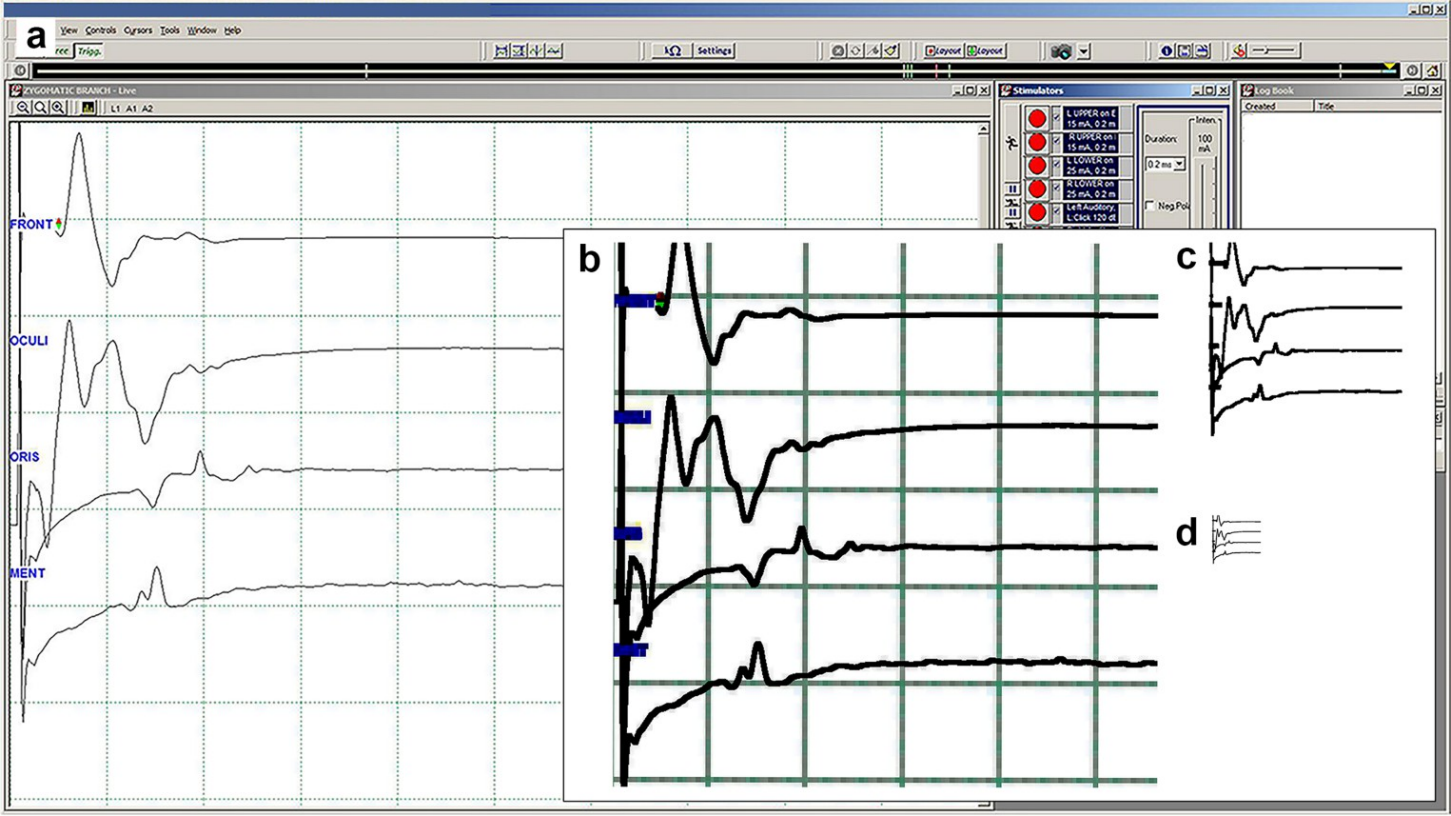
Image preparation. Setting the region of interest and removing unnecessary data other than the graph itself. The original screenshots were 1,920 × 1,080 pixels (width x height), with an RGB color profile. We cropped the images (B) to 720 × 720 pixels, ensuring they contained all four graphical waves of the facial muscles from the moment of facial nerve stimulation to the disappearance of electrical activity. Next, a minimum filter was applied to thicken and enhance the line art information. After resizing the image to 256 × 256 pixels, converting them to black and white, and removing the background information and grid (C), we resized the images to 64 × 64 pixels (D).

The goal of data preprocessing was to set the region of interest and remove unnecessary data other than the graph itself. The original screenshots measured 1,920 × 1,080 pixels (width × height) and had an RGB color profile. We cropped the images to 720 × 720 pixels, ensuring they contained all four graphical waves of the facial muscles from the moment of facial nerve stimulation to the disappearance of electrical activity. Next, a minimum filter was applied to thicken and enhance the line art information. After resizing the images to 256 × 256 pixels, converting to black and white, and removing the background information and grid, we resized the images to 64 × 64 pixels (Fig 2).

### Deep neural network implementation

The neural networks were constructed by combining several convolutional, average pooling, and dense layers. We trained the neural networks using an ADAM optimizer [11] and binary cross-entropy as the loss function, which performs as well as the current-standard, off-the-shelf, open-source software and common hardware. To improve performance, we trained the neural networks using more than 2,500 patterns of hyper-parameter variable combinations, such as convolutional layer depth, the number of convolutional layer filters, dense layer size, batch size, optimizer learning rate, and regularizer settings for each layer, among others. We added early stopping and L2 regularization functions to avoid overfitting [12–14].

### Evaluation of classification results

We prepared a completely new set of images to test the model, 25 with LSR and 25 without LSR, from another 25 patients whose data had not been used at all in training or validation. Therefore, the images in the test had never been exposed to the neural network.

Among the models built with combinations of hyper-parameter variables, those with the highest accuracy and area under the receiver operating characteristic curve (AUC) when classifying the test set were compared with the assessments of neurosurgeons and IOM technologists experienced in HFS and microvascular decompression. To ensure that performance was compared between the neural network and human participants under the same conditions, the experts did not have access to the patients’ clinical data, such as demographic characteristics, the patient’s preoperative condition, or stage of surgical procedure at the time of test image acquisition. The following metrics were used to evaluate the classification performance of the neural network and human professionals: primary outcome of classification accuracy, calculated as the proportion of images in the test set that were labeled correctly, and common metrics such as the sensitivity, specificity, positive and negative predictive values, and AUC.

### Heatmaps

Gradient-weighted class activation maps (Grad-CAM++) [15] and saliency maps that applied the SmoothGrad method [16] were used to visualize the focus made by the neural network. This process was performed using the Python tf-keras-vis toolkit (https://github.com/keisen/tf-keras-vis), which was contributed by one of the authors (Y.K.). These methods use localization maps that visualize the spatial information of the network input in the convolutional layers and expand them to an input-sized saliency map.

### Packaging into a computer-assisted interpreting program

The best performing neural network was packaged into a standalone application running on a 64-bit Windows system without Python installed. This process was performed using the Pyinstaller module (https://www.pyinstaller.org).

### Statistical analysis

Predictive accuracy is expressed as mean ± standard deviation. Differences between groups were analyzed using one-sample *t*-tests. All *p*-values <0.05 were considered statistically significant. All data analyses were conducted using GraphPad Prism version 6.01 for Windows (GraphPad Software, La Jolla, CA, USA).

## Results

### The neural network could accurately classify images of intraoperative EMG with LSR presence

The neural network that we constructed was successful in correctly differentiating all 50 test images. The sensitivity, specificity, and positive and negative predictive values were calculated as 100%. The neural network with the best accuracy and AUC was constructed by combining the following hyper-parameter settings: depth of convolutional layers, 5; number of convolutional layer filters, 32; batch size for fitting, 8; learning rate of optimizer, 0.0003. The structure of the neural network is shown in Table 1.

**Table 1:** The constructed neural network.

Layer	Type of layer
Input (64 × 64, black and white)	
Conv1	Conv 2D-32
Pool	Average pooling 2D
Conv2	Conv 2D-64
Pool	Average pooling 2D
Conv3	Conv 2D-128
Pool	Average pooling 2D
Conv4	Conv 2D-256
Pool	Average pooling 2D
Conv5	Conv 2D-512
GlobalPool	Global average pooling 2D
Output	Dense

### The neural network and human professionals showed equivalent performance

The images were classified by the human participants under the same conditions as the neural network. The performance of the neural network was compared with that of 16 human professionals (11 neurosurgeons and 5 IOM technologists). All 11 board-certified neurosurgeons were currently active in the field of functional neurosurgery and were trained in microvascular decompression surgery. Six of them had more than 3 years of experience in their functional neurosurgical field. All 5 IOM technologists performed neurophysiological monitoring in the operation room during the neurosurgical or orthopedic procedures. Four of them had more than 3 years of experience in neurophysiological monitoring.

**Fig 3:**
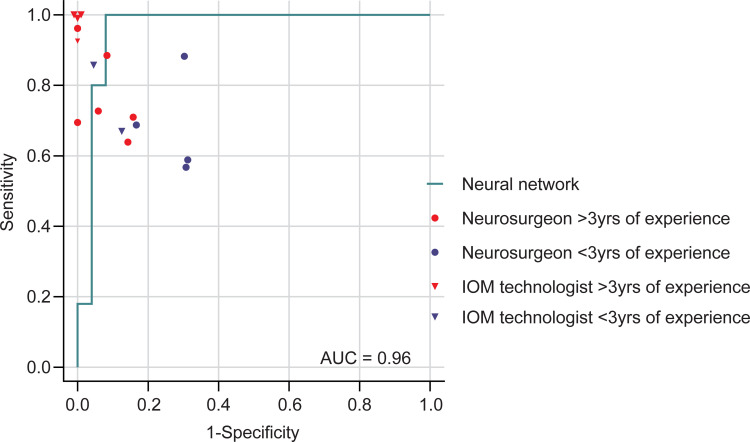
Receiver operating characteristic curve. A professional with more than 3 years of experience tended to be more accurate than a less experienced professional in classifying lateral spread response (LSR), but the difference in accuracy was not statistically verifiable between the neural network and subgroups by experience due to the small number of participants included. To classify 50 images as LSR presence or absence, the neural network’s AUC was 0.96. AUC, area under the receiver operating characteristic curve; IOM, intraoperative monitoring.

The proposed neural network obtained 100% accuracy, which was significantly higher (*p* = 0.001) than that of human professionals (average, 82.8% ± 13.6%). When we categorized the human professionals according to their experience and specialty, the accuracy of the technologist group (94.0% ± 11.3%) was significantly higher (*p* = 0.0200) than that of the neurosurgeon group (77.7% ± 11.7%). The performance of the neural network was equivalent to that of the technologist group (*p* = 0.3013) but better than that of the neurosurgeon group (*p* < 0.0001). A professional with more than 3 years of experience tended to be more accurate at classifying the LSR than a professional with less experience, but the difference in accuracy was not significant between the neural network and experience subgroups. In the binary classification of the 50 images of LSR presence or absence, the AUC of the neural network was 0.96 (Fig 3).

### The trained neural network identified key features differentiating LSR findings

**Fig 4:**
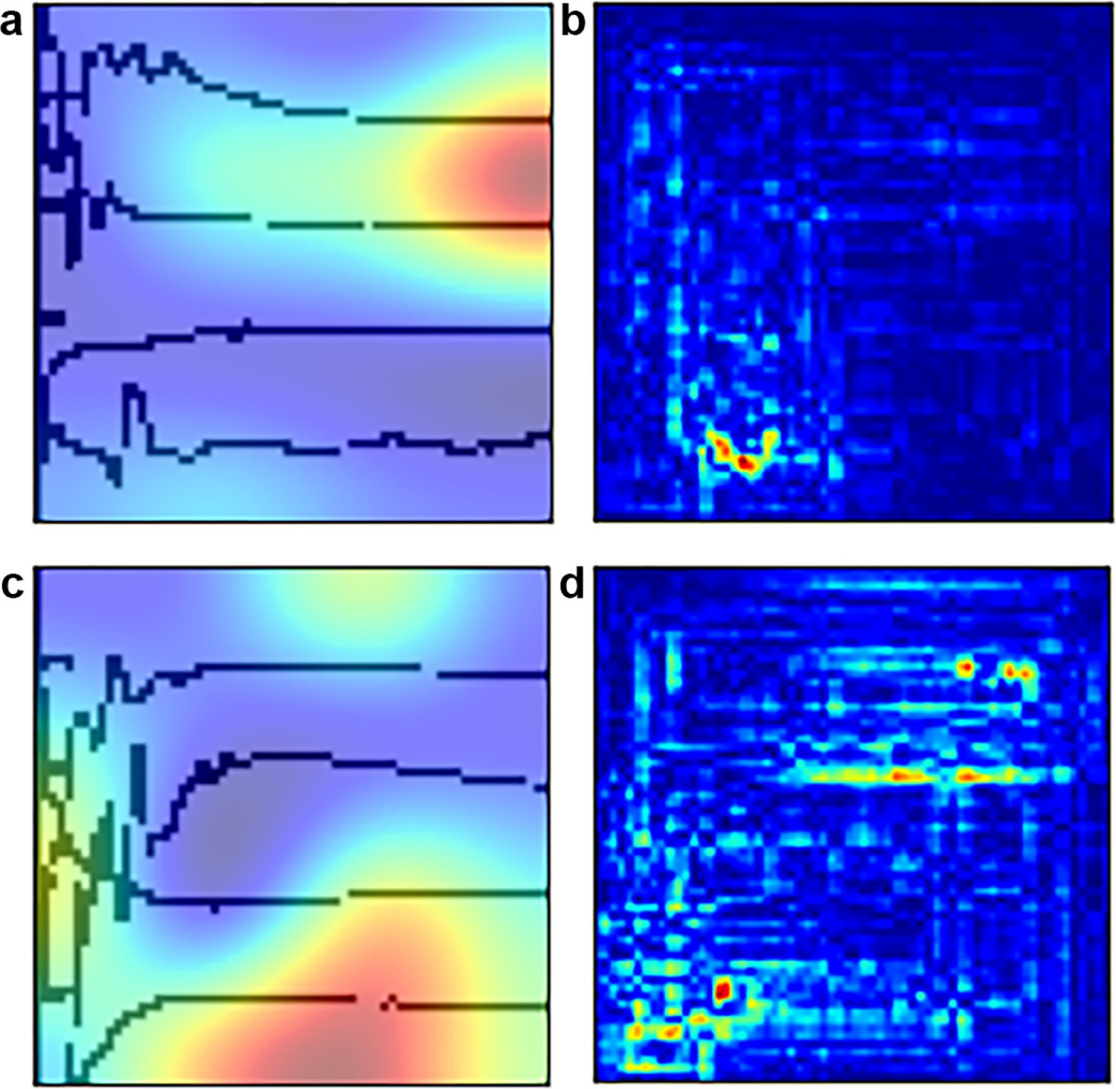
Heatmaps of the representative lateral spread response (LSR) images. Increased intensity was observed around the lowermost wave, representing the response of the mentalis muscle in the images with LSR presence (A, B) and absence (C, D). (A, B) In those with LSR presence, the presence of the peak of the lowermost wave is the main criterion for judgment, as can be seen in the hot spots, especially in (B). (C, D) In the case of LSR absence, the deep neural network concentrates the entire lowermost wave because there is no peak in the corresponding region. This finding is very similar to how humans interpret graphs. Red indicates the focus of the neural network.

The heatmap of the representative LSR images is shown in Fig 4. Increased intensity was observed around the lowermost wave, representing the response of the mentalis muscle; this was identified as the key feature in differentiating LSR presence and absence. In images with LSR presence, hot uptake was apparent near the peak of the lowermost wave, whereas hot uptake in images with LSR absence was spread all over the lowermost wave. This finding illustrates why our neural network could correctly classify the test images. The heatmap images highlighted the same area that humans read when interpreting the EMG graphs to classify the LSR. Among the two visualization techniques, the SmoothGrad images best displayed the region that our neural network might have utilized for its inference.

### The neural network-based application was useful in real circumstances

In clinical practice, neural networks are used to assist clinicians in the classification of new observations into one of the labeled groups. In the present study, this meant recognizing whether the intraoperative EMG wave had an LSR or not. The neural network was packaged into an executable binary program available for 64-bit Windows; thus, it would return a prediction if exposed to images. The program does not require training, nor does it need a computer with a graphic processing unit. After exposure to the image, it rapidly displays the prediction on the screen within a second.

We tentatively tested the program in a clinical situation. Although our test could not be validated statistically or officially published in its current setting, it may be useful, especially when the LSR findings differed from those of the IOM technologist. In such cases, the IOM technologist notified the surgeon about the discrepancy of the finding and reported both their own and the neural network’s prediction. The surgeon was therefore informed and could interact better with the IOM technologist about the situation.

## Discussion

This study showed that deep learning algorithms for classifying LSR presence and absence can perform well. The network provided accuracy that was equivalent to or better than that of the human professionals for classification (Fig 3). It could be used to provide clinical support during surgery when IOM findings are insufficient for concise classification by a human. Interestingly, the heatmap generated by the Grad-CAM++ and SmoothGrad algorithms highlighted the key region of interest within each image at a level similar to that of trained human professionals.

Our most important contribution was that we built a neural network to classify a line chart. Most previous publications have used deep learning algorithms to categorize medical photos [17–20], radiographic images [21–25], pathologic specimens [26–28], or other microscopic features [29, 30] that often require labor-intensive preprocessing before being input into a neural network. Conversely, our network could use screenshotted information immediately acquired from the IOM suite, without any time-consuming preprocessing of the data. Interpreting medical information via a line chart image, without the need for calculation, is more similar to what humans actually do, and the heatmap in Fig 4 supports that assumption. The neural network focused only on the peak of the lowermost wave, which represented an abnormal latent response in the mentalis muscle in the image with LSR presence (Fig 4A and 4B). Conversely, the neural network seemed to search for the peak on the whole lowermost wave of the image with LSR absence (Fig 4C and 4D).

Neural networks table IOMs can give prompt feedback to the surgical team during operations. Our algorithms can recognize the presence or absence of the LSR immediately after the EMG graph appears on the screen of the IOM suite. Thus, real-time feedback to the surgical team serves the purpose of the IOM itself. That is, surgeons can gain information both from the IOM technologist and our algorithm. Sometimes, mismatched information between interpreters and machine learning can prompt surgeons to reconsider the adequate localization for decompression of the target nerve. As shown in Fig 3, the accuracy of interpretation by an inexperienced interpreter was inferior to machine learning. Therefore, if the surgical team is inexperienced or if human error is possible due to fatigue, it would be preferable to use machine learning as an auxiliary device.

The present study has some limitations. First, only 3,600 images of 50 participants from a single center were included in the training and validation sets. However, the neural network showed good performance when tested using completely new data from 25 participants. This could also be interpreted as an advantage; a neural network can be created using a small sample size of data at each individual institute. Second, the screen layout for IOM monitoring varies greatly depending on user preference and manufacturer design. To overcome this limitation, we have developed a standalone application that runs on a 64-bit Windows-based system. The neural network should return the right answer as long as the captured position is adjusted to suit the screen layout of the equipment. The present study, therefore, overcame one major limitation of previous studies, namely that deep learning algorithms in prior clinical applications have rarely been applied outside the original study. Finally, essential clinical information that should be considered when reading an EMG was excluded from the present study. In further studies, we will add information such as time elapsed from the start of the operation and temporal changes from the initial image to ensure that the neural network has similar information as that when a human makes a judgment.

In conclusion, the present study showed that a deep neural network trained on a relatively small dataset of intraoperatively collected EMGs could detect LSR, a characteristic pathology in patients with HFS, and that the performance of the neural network was similar to that of human neurosurgeons and IOM technologists. These results were obtained from medical line chart information using standard, off-the-shelf, open-source software and common hardware. Therefore, our deep learning algorithm may be useful to surgical teams during operations.
